# Comparison of Renal Stones and Nephrocalcinosis in Children: Findings From Two Tertiary Centers in Saudi Arabia

**DOI:** 10.3389/fped.2021.736308

**Published:** 2022-01-17

**Authors:** Khalid A. Alhasan, Mohamed A. Shalaby, Amr S. Albanna, Mohamad-Hani Temsah, Zainab Alhayek, Mohammed S. Abdalla, Najlaa G. Alotaibi, Nada M. Kalakattawi, Zaher Faisal Zaher, Jameela A. Kari

**Affiliations:** ^1^Pediatrics Department, College of Medicine, King Saud University, Riyadh, Saudi Arabia; ^2^Pediatrics Department, King Saud University Medical City, Riyadh, Saudi Arabia; ^3^Pediatric Nephrology Center of Excellence, Department of Pediatrics, King Abdulaziz University, Jeddah, Saudi Arabia; ^4^Department of Pediatrics, King Abdulaziz University, Jeddah, Saudi Arabia; ^5^King Abdullah International Medical Research Center, King Saud Bin Abdulaziz University for Health Sciences, Jeddah, Saudi Arabia

**Keywords:** children, nephrolithiasis, urolithiasis, outcomes, renal stones

## Abstract

**Background:** Renal stones (nephrolithiasis and urolithiasis) and nephrocalcinosis are uncommon in children; however, their incidences in pediatric populations have been increasing.

**Patients and Methods:** This multicenter retrospective study compared the clinical presentation, etiology, and outcomes of childhood nephrolithiasis or urolithiasis with those of nephrocalcinosis.

**Results:** The study included 144 children: 93 with renal stones and 51 with nephrocalcinosis. The mean age at presentation was 72 months and 54 months for children with renal stones and nephrocalcinosis, respectively. A history of consanguinity was found in 65% and 76% of the cases of renal stones and nephrocalcinosis, respectively. Congenital anomalies of the kidneys and urinary tract (CAKUT) were present in 28 and 9.8% of the patients with renal stones and nephrocalcinosis, respectively. The most common symptoms of renal stones were flank pain (29%), hematuria (15%), and dysuria (11%). Urinary tract infection was the primary presentation in the nephrocalcinosis group (18%), followed by failure to thrive (16%), polyuria (12%), and dehydration (12%). The majority of renal stone cases were caused by metabolic disorders, including hyperoxaluria (18%), cystinuria (18%), hypercalciuria (12%), and hyperuricosuria (2%). In contrast, the most common underlying disorders in cases of nephrocalcinosis were familial hypomagnesemia, hypercalciuria, nephrocalcinosis (35%), distal renal tubular acidosis (23%), and Bartter syndrome (6%). Clinical outcomes were significantly better in children with nephrolithiasis/urolithiasis than in those with nephrocalcinosis, who showed radiological evidence of worsening/persistent calcinosis and progressed more frequently to chronic kidney disease (stage II-IV) and end-stage kidney disease.

**Conclusion:** The average age at presentation for children with renal stones was greater than that for those presenting with nephrocalcinosis. More than 25% of the children with renal stones were found to have CAKUT. Nephrocalcinosis was associated with worse clinical outcomes related to kidney function and disease resolution than nephrolithiasis.

## Introduction

Nephrolithiasis refers to the presence of stones within the renal pelvis, while urolithiasis refers to stones in the kidney that are localized within the tubular lumen and lower urinary tract or primary bladder stones (1, 2). Nephrocalcinosis is characterized by deposition of calcium salts in the renal parenchyma. Nephrocalcinosis is classified ultrasonographically into cortical, medullary, and diffuse nephrocalcinosis. All three entities (nephrolithiasis, urolithiasis, and nephrocalcinosis) are relatively uncommon in the pediatric population; however, cases of these entities in children are increasing (3) and are becoming increasingly common causes for hospital admission or visits to renal clinics. In addition, all of these conditions are associated with significant long-term sequelae, including morbidity caused by recurrent stones as well as the development of chronic kidney disease (CKD) and renal impairment.

The annual incidence of pediatric nephrolithiasis has increased from 6 to 10% over the past 20 years in the United States (3), with the greatest increase observed among adolescent black girls (4). The etiology of this condition is metabolic in most children, with hematuria and urinary tract infections being the most common presentations (5). However, the clinical presentation varies with age, with flank pain appearing in older children or adolescents and more vague symptoms such as nausea, vomiting, and irritability typically appearing in younger children. Incidental findings on imaging studies have also been reported in a considerable proportion of affected children (6).

Kidney stone formation is caused by urine with a solute concentration higher than its solubility (3). The imbalance of promoters and inhibitors causes crystallization, and the attachment and growth of crystals into nephroliths occurs due to epithelial abnormalities (7). The management of pediatric nephrolithiasis includes urinary decompression, medical treatment of specific risk factors, and surgical intervention (8).

In this study, we have compared the epidemiology, etiology, and outcomes of children presented with nephrolithiasis urolithiasis with that of nephrocalcinosis from two tertiary centers in the Kingdom of Saudi Arabia (KSA) as the etiologies are different but these entities are closely related, one may form in the apparent absence of the other (9). Traditionally, nephrolithiasis and nephrocalcinosis have been thought to result from systemic conditions and their formation depends upon both the delivery of minerals such as calcium, phosphate and oxalate, and local factors such as pH, osmolality, and the relative absence of a variety of inhibitory molecules and proteins we think also that a little known about the difference in the outcomes.

## Patients and Methods

### Study Design

This multicenter retrospective study was conducted at two tertiary centers in KSA (King Abdulaziz University Hospital and King Saud University Medical City).

### Patients and Evaluations

Children diagnosed with renal stones or nephrocalcinosis were included and followed up at the recruiting pediatric nephrology units over a period of 4 years (between January 2015 and December 2018). The inclusion criteria were age 14 years or younger and a radiologic diagnosis of renal stones and nephrocalcinosis.

Renal stones were defined by the presence of a stone in two images, excluding artifacts. Children showing spontaneous passage of a stone and those who underwent removal of a stone by surgical intervention were included if stone analysis was available. We used ultrasound or computed tomography (CT) to detect faint calcifications. On the basis of renal ultrasound findings, medullary nephrocalcinosis was classified as follows: mild (early hyperechogenicity in the periphery of the pyramids), moderate (diffuse hyperechoic pyramids), and severe (clumps of renal pyramids) (10).

All demographic and clinical data were collected from the patients' electronic hospital records and included age at presentation, sex, creatinine level, estimated glomerular filtration rate (eGFR) at presentation, medical and surgical history, presence of a family history of nephrolithiasis or CKD, consanguinity, symptoms and signs at presentation, number and localization of stones, and grades of nephrocalcinosis. All patients presenting with symptoms and/or signs of urinary tract infection (UTI) were screened using urine analysis and urine culture. Urine samples were collected as midstream for toilet trained children or by transurethral catheterization for non-toilet trained children. UTI was defined by the presence of more than five white blood cells per high-power field (hpf) and a positive urine culture with more than 100,000 bacterial colony counts per ml. The estimated glomerular filtration rate had been calculated using Schwartz formula (11).

To diagnose metabolic renal stones or nephrocalcinosis, we combined the data obtained from urine metabolic work-up, genetic tests with the relevant clinical data, and stone analysis. Metabolic workup of nephrolithiasis was performed using a spot urine sample and was interpreted in terms of the solute/creatinine ratio (12). The test was repeated twice to confirm hypercalciuria, hyperoxaluria, cystinuria, hypocitraturia, and hyperuricosuria. Hypercalciuria, hyperoxaluria, and hyperuricosuria were defined by urinary solute:creatinine ratios greater than the 95th percentile as a function of age (Table 1). We also examined the associated acid-base and electrolyte disturbances such as metabolic acidosis and alkalosis, hypo-or hypernatremia, hypo-or hyperkalemia, hypo- or hyperchloremia, hypo- or hypercalcemia, and hypo- or hyperphosphatemia. Hypernatremia and hyponatremia were defined by serum sodium levels >145 mmol or <35 mmol, respectively. Hyperkalemia was defined by a serum potassium level > 5.5 meq/L in children and >6 meq/L in neonates, and hypokalemia was defined by a potassium level <3.5 meq/L. Hypercalcemia was defined by a serum calcium level greater than 2.6 mmol/L. Acid base disturbance was tested using venous sample, metabolic acidosis was defined by pH <7.35 and serum bicarbonate level <18 meq/L, whereas metabolic alkalosis was defined by pH > 7.45 and serum bicarbonate level > 25 meq/L.

**Table 1:** Cut-off levels of solute:creatine ratios in relation to age group (12).

**Item**	**Range (month)**	**Range (year)**	**Normal value**
Ca/creatine (mmol/mmol)	0–12	0-1	2.2
	12–36	1-3	1.5
	36–60	3-5	1.1
	60–84	5-7	0.8
	>84	>7	0.6
Oxalate/creatine (mmol/mmol)	0–12	0-1	0.17
	12–24	1-2	0.13
	24–36	2-3	0.1
	36–60	3-5	0.08
	60–84	5-7	0.07
	>84	>7	0.06
Cystine/creatine (mmol/mol)	0–1	0->1	85
	1–6		53
	>6		18
Citrate/creatine (mmol/mmol)	0–60	0-5	0.12
	>60	>5	0.08
Uric acid/creatinine (mmol/mmol)	0–12	0-1	1.5
	12–36	1-3	1.3
	36–60	3-5	1
	60–120	5-10	0.6
	>120	>10	0.4

Renal imaging (renal ultrasound, X-ray, and CT) data were reviewed and used for classification, as previously mentioned, our protocol was to do ultrasound only, and CT scan to be done if urology colleagues requesting it before stone procedure like ESW or in case patients having persistence symptoms with normal ultrasound and we are suspecting ureteral stone.

Genetic tests and stone analysis were performed when possible and were used as confirmatory tests to diagnose the underlying causes of kidney disease; genetic testing has been done for all patients with primary hyperoxaluria and familial hypomagnesemia hypercalciuria nephrocalcinosis. But was not done in all cases of cystinuria and barters, in which diagnosis was depends on metabolic work up and clinical manifestation in addition to stone analysis for cystinuria.

All confirmed cases of renal stones or nephrocalcinosis were followed up, and serial imaging studies (renal ultrasound and/or X-ray and/or helical CT) were performed to evaluate the outcome. Estimated glomerular filtration rate (eGFR) was calculated using the Schwartz formula (13). All clinical data about the stones, such as the number, location, laterality, and grades of nephrocalcinosis were collected. All associated congenital anomalies of the kidney and urinary tract were reported. Stone analysis was performed for patients who spontaneously passed their stones. We documented the treatment modalities used, which included conservative treatment, extracorporeal shock wave lithotripsy (ECSL), or surgical therapies.

The outcomes of renal stones (nephrolithiasis/urolithiasis) or nephrocalcinosis were monitored and categorized as follows: spontaneous resolution, post-intervention resolution, persistence, worsening or new stone formation, or nephrocalcinosis. Cases that were missed during follow-up were identified and documented. Spontaneous remission was defined by the disappearance of stones and/or nephrocalcinosis in two or more serial imaging studies. The renal outcome was used as an indicator of morbidity and was assessed by measuring the reduction in eGFR in comparison with the initial eGFR and determining the presence and severity of proteinuria. The incidence of mortality was also reported.

### Statistical Analysis

All analyses were performed using STATA (StataCorp. 2011. Stata Statistical Software: Release 12. College Station, TX, StataCorp LP). The proportion and mean for dichotomous and continuous variables, respectively, were measured to describe patient characteristics. Comparative analyses were performed using the chi-square test for categorical variables, and Student's *t*-test was performed for continuous variables. Statistical significance was determined using a 95% confidence interval and a *p*-value of 0.05.

## Results

We identified 144 patients who met the inclusion criteria (93 presented mainly with nephrolithiasis/urolithiasis and 51 with nephrocalcinosis). The mean age at presentation for children with nephrolithiasis was greater than that for children with nephrocalcinosis (mean age, 72 months vs. 54 months). Most patients with nephrolithiasis were male (66.7%), with a male-to-female ratio of 2:1. In contrast, nephrocalcinosis occurred mainly in female patients (60.4%), with a male-to-female ratio of 2:3. Furthermore, a greater proportion of children with nephrolithiasis had normal kidney function at the time of presentation. The mean eGFR at the time of presentation was higher in children with nephrolithiasis (148.4 vs. 122.72 ml/min/1.73 m^2^). A history of consanguinity was found in 64.8% of patients with nephrolithiasis (56% of the patients had parents who were first-degree cousins). A 23.1% of this group had a family history of nephrolithiasis, and a 36.3% had a family history of CKD (Table 2). In the nephrocalcinosis group, consanguinity was found in 76% of the cases. Of these, 35% had a family history of nephrolithiasis and 23.7% had a family history of CKD (Table 2). Congenital anomalies of the kidney and urinary tract were reported in 28% of children with renal stones and in 9.8% of those with nephrocalcinosis (Table 2).

**Table 2:** Patients' baseline demographic and disease characteristics.

**Characteristics**	**Renal stones**		**Nephrocalcinosis**	
	**Estimate**	**95% CI**	**Estimate**	**95% CI**
Age at presentation (mean, month)	72.22	62.7–84.7	54.25	39.1–63.6
Male sex (%)	66.7	56.6–75.7	39.2	26.6–53.0
Creatinine (mean, at presentation)	60.24	39.6–80.7	83.90	41.2–126.5
eGFR (mean, at presentation)	148.31	136.2–160.3	122.72	104.4–140.9
Consanguinity (%)	64.8	54.6–74.1	78.7	65.3–88.7
Family history of nephrolithiasis	23.1	15.3–32.6	59.6	45.2–72.8
Family history of renal disease	36.3	26.9–46.5	51.1	33.7–60.7
CAKUT (%)	28	23.0–44.6	9.80	0.3–20.3

*eGFR, estimated glomerular filtration rate in ml/min/1.73m^2^; CAKUT, congenital anomalies of the kidneys and urinary tract; ESRD, end-stage renal disease*.

**Figure 1:**
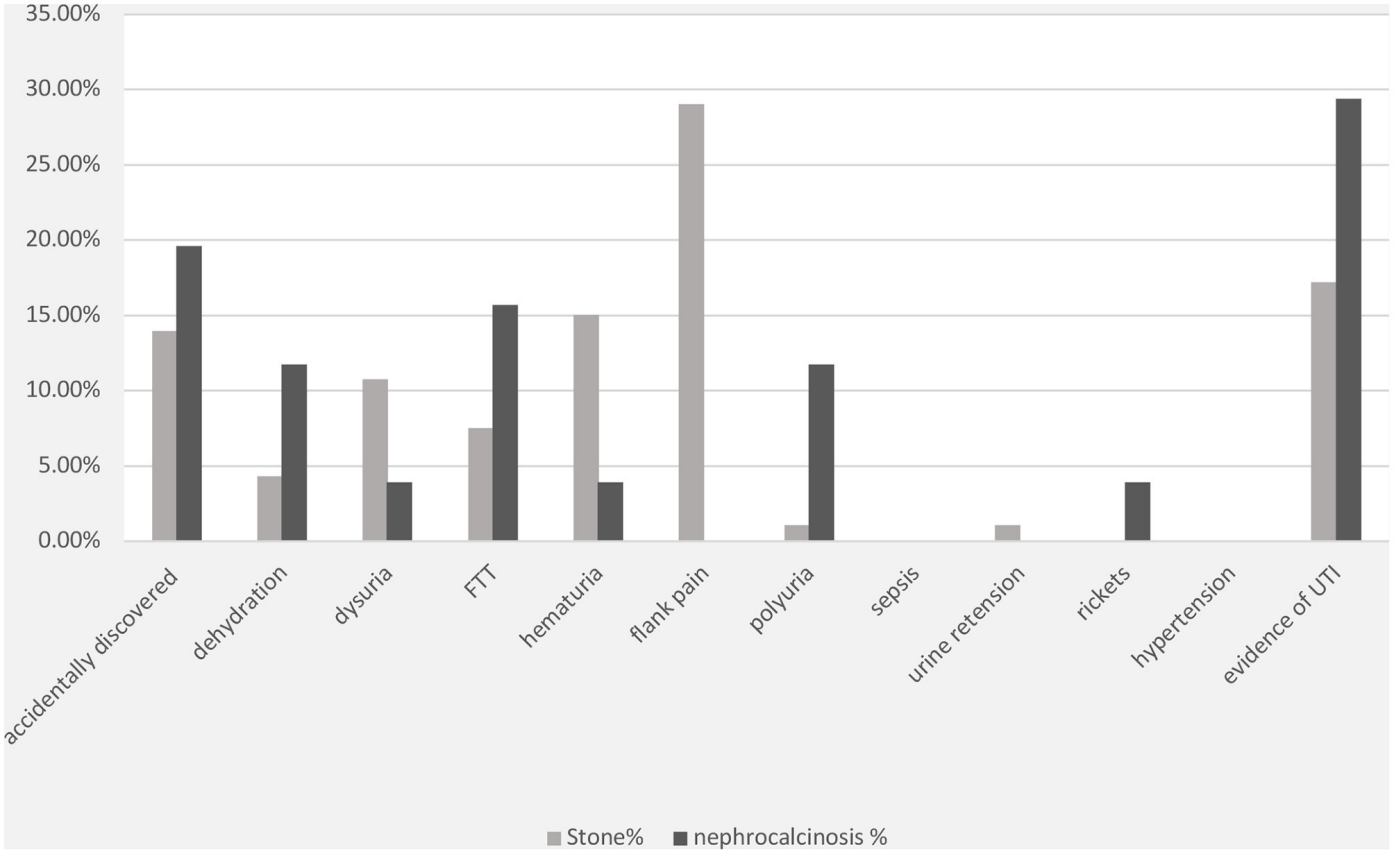
Clinical presentations of patients with nephrolithiasis/urolithiasis (renal stone) and nephrocalcinosis. FTT, failure to thrive; UTI, urinary tract infection; FHHNC, familial hypomagnesemia hypercalciuria nephrocalcinosis.

The most frequent presenting symptom of nephrolithiasis was flank pain (29%), followed by hematuria (15%) and dysuria (11%). Approximately 19% of the children presented with signs and symptoms of UTI, which was confirmed by urine culture in 15% of the cases. Nephrolithiasis was incidentally discovered during routine investigations in 14% of the patients. Other symptoms such as failure to thrive, dehydration, and polyuria were reported less frequently (Figure 1). UTI symptoms and signs were the main presentation in the nephrocalcinosis group (31%); however, UTI was confirmed with a urine culture in only 18% of the cases. Failure to thrive, polyuria, and dehydration were the presenting symptoms in 16, 12, and 12%, respectively, of the children with tubulopathy and nephrocalcinosis (Bartter syndrome and distal renal tubular acidosis). Nephrocalcinosis was incidentally diagnosed during routine investigations in 18% of the children. Other less frequent presentations included rickets, dysuria, and hematuria (Figure 1).

**Figure 2:**
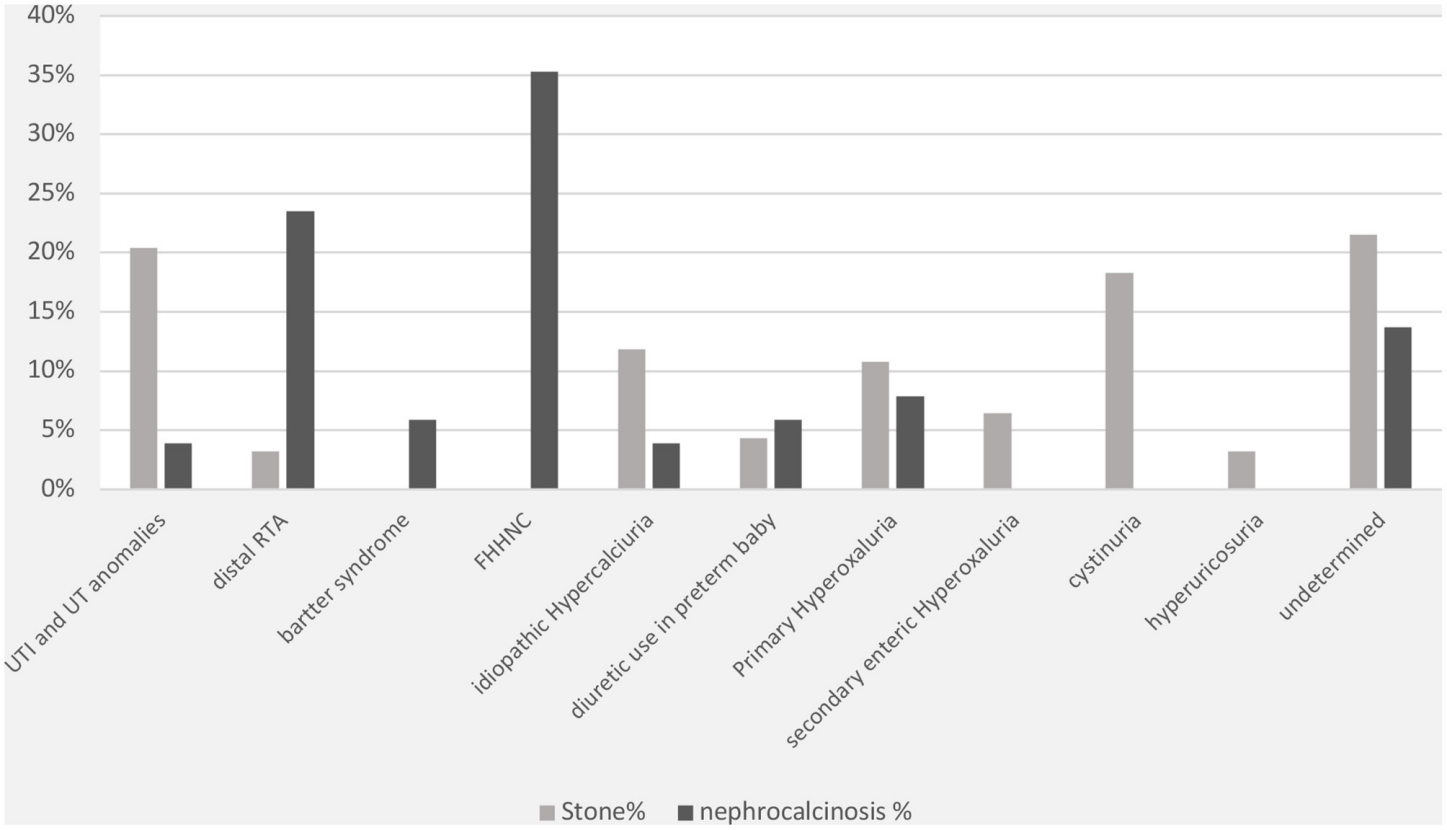
Comorbidities associated with nephrolithiasis/urolithiasis (renal stone) and nephrocalcinosis. FHHNC, familial hypomagnesemia hypercalciuria nephrocalcinosis.

The specific risk factors were identified in 78% of the cases, whereas it was idiopathic in 22% of the children. Metabolic disorders were observed in 69% of the cases and included the following conditions: hyperoxaluria (18%), cystinuria (18%), hypercalciuria (12%), and hyperuricosuria (2%). UTI was documented in 20% of cases. Distal renal tubular acidosis and diuretic use were reported in <5% of the children in this group. In contrast, the underlying cause of nephrocalcinosis was determined in 86% of the cases (familial hypomagnesemia hypercalciuria nephrocalcinosis [FHHNC], 35%; distal renal tubular acidosis, 23%; and Bartter's syndrome, 7%) (Figure 2). The results for urinary metabolites in terms of solute/creatine ratio and serum electrolytes for both the renal stone and nephrocalcinosis groups are presented with the corresponding 95% confidence intervals (CIs) in Tables 3, 4.

**Table 3:** Urine chemistry results among patients with nephrolithiasis/urolithiasis (renal stones) and nephrocalcinosis.

**Characteristics**	**Renal stones**		**Nephrocalcinosis**		***P*-value**
	**Estimate**	**95% CI**	**Estimate**	**95% CI**	
Recurrent UTI (%)	36.71%	25.8–47.6	17.24%	0.3–20.3	0.09
Ca/creatinine (mean, mmol/mmol)	1.35	0.7–2.1	2.07	1.3–2.7	0.168
Oxalate/creatinine (mean, mmol/mmol)	2.18	0–4.9	7.12	0–19.3	0.433
Cystine/creatinine (mean, mmol/mmol)	29.47	17.2–41.6	5.28	0–11.7	0.408
Citrate/creatinine (mean, mmol/mmol)	6.86	4.7–8.9	8.63	4.2–13.1	0.449
Uric acid/creatinine (mean, mmol/ mmol)	0.63	0.4–0.8	0.74	0.3–1.1	0.718
FEMg% (mean)	2.07	1.7–2.3	8.78	5.7–11.7	<0.001
TRP% (mean)	91.82	90.8–92.0	87.76	84.4–91.0	0.02

*CI, confidence interval; FEMg%, fraction of excretion of magnesium; TRP, tubular reabsorption of phosphate %; UTI, urinary tract infection*.

**Table 4:** Serum electrolyte results among patients with nephrolithiasis/urolithiasis (renal stones) and nephrocalcinosis.

**Characteristics**	**Renal stones**		**Nephrocalcinosis**		***P*-value**
	**Estimate**	**95% CI**	**Estimate**	**95% CI**	
Serum sodium (mean)	139.04	138.2–139.8	139.43	138.3–140.4	0.567
Serum K (mean)	4.15	4.04–4.25	3.78	3.58–3.95	0.001
Serum chloride (mean)	103.66	102.83–104.46	103.18	101.37–104.97	0.58
pH (mean)	7.37	7.36–7.37	7.38	7.33–7.42	0.505
Serum HCO_3_ (mean)	23.59	23.15–24.16	25.06	23.53–26.58	0.086
Serum Ca (mean)	2.38	2.35–2.40	2.28	2.21–2.32	0.001
Serum phosphate (mean)	1.57	1.49–1.62	1.47	1.32–1.59	0.184
Serum Mg (mean)	0.83	0.63–1.02	0.73	0.67–0.76	<0.001

**Figure 3:**
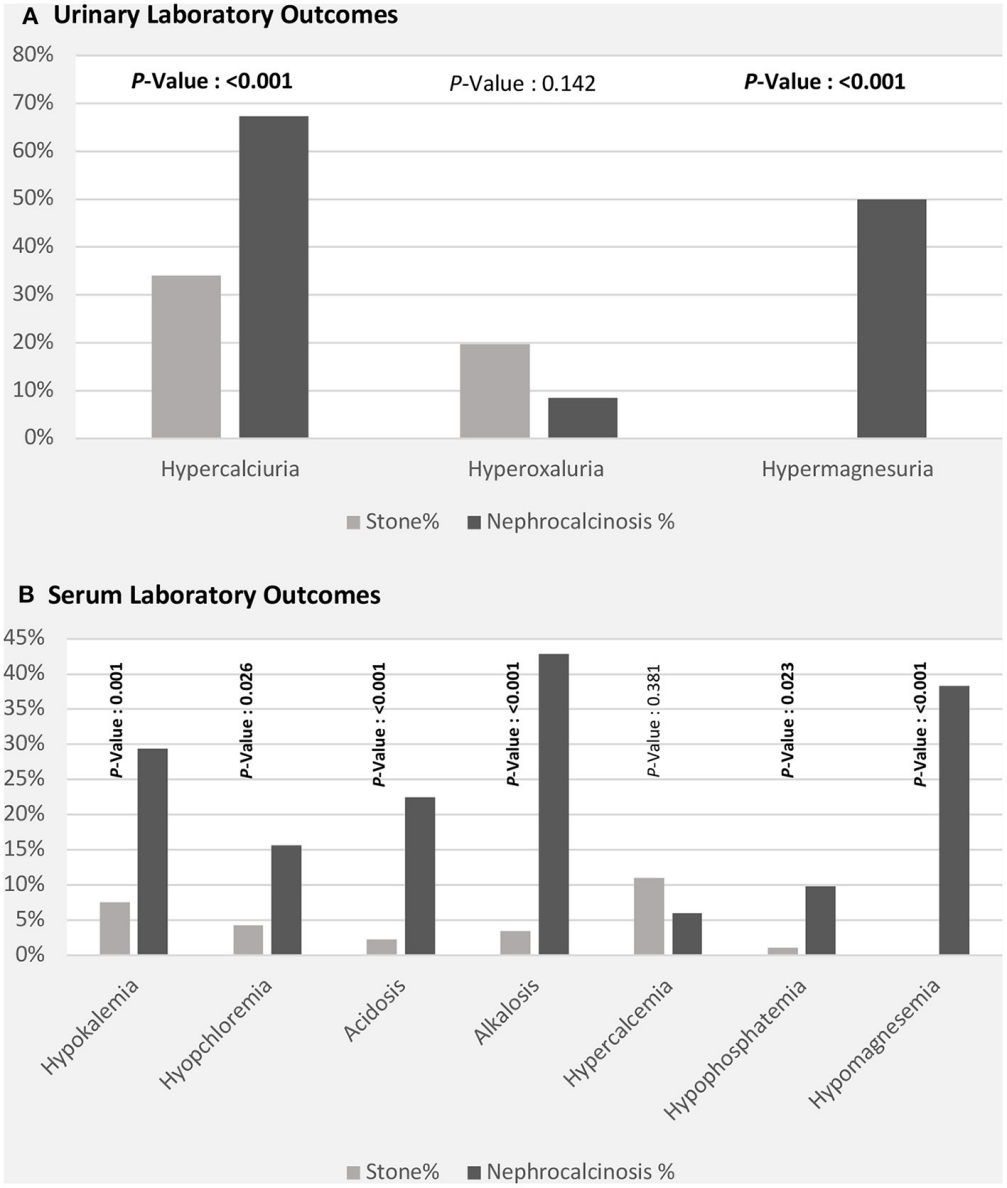
Laboratory outcomes [urinary **(A)** and serum **(B)**] among patients with nephrolithiasis/urolithiasis (renal stone) and nephrocalcinosis.

The high frequency of FHHNC explains the higher incidences of hypermagnesuria, which was represented as fractional excretion of magnesium (FEMg%), and hypercalciuria in the nephrocalcinosis group in comparison with the nephrolithiasis group (*p* ≤ 0.001; Figure 3A). Similarly, the incidences of hypokalemia, hypomagnesemia, and acid-base disturbance were significantly higher in the nephrocalcinosis group (*p* < 0.001, Figure 3B).

**Figure 4:**
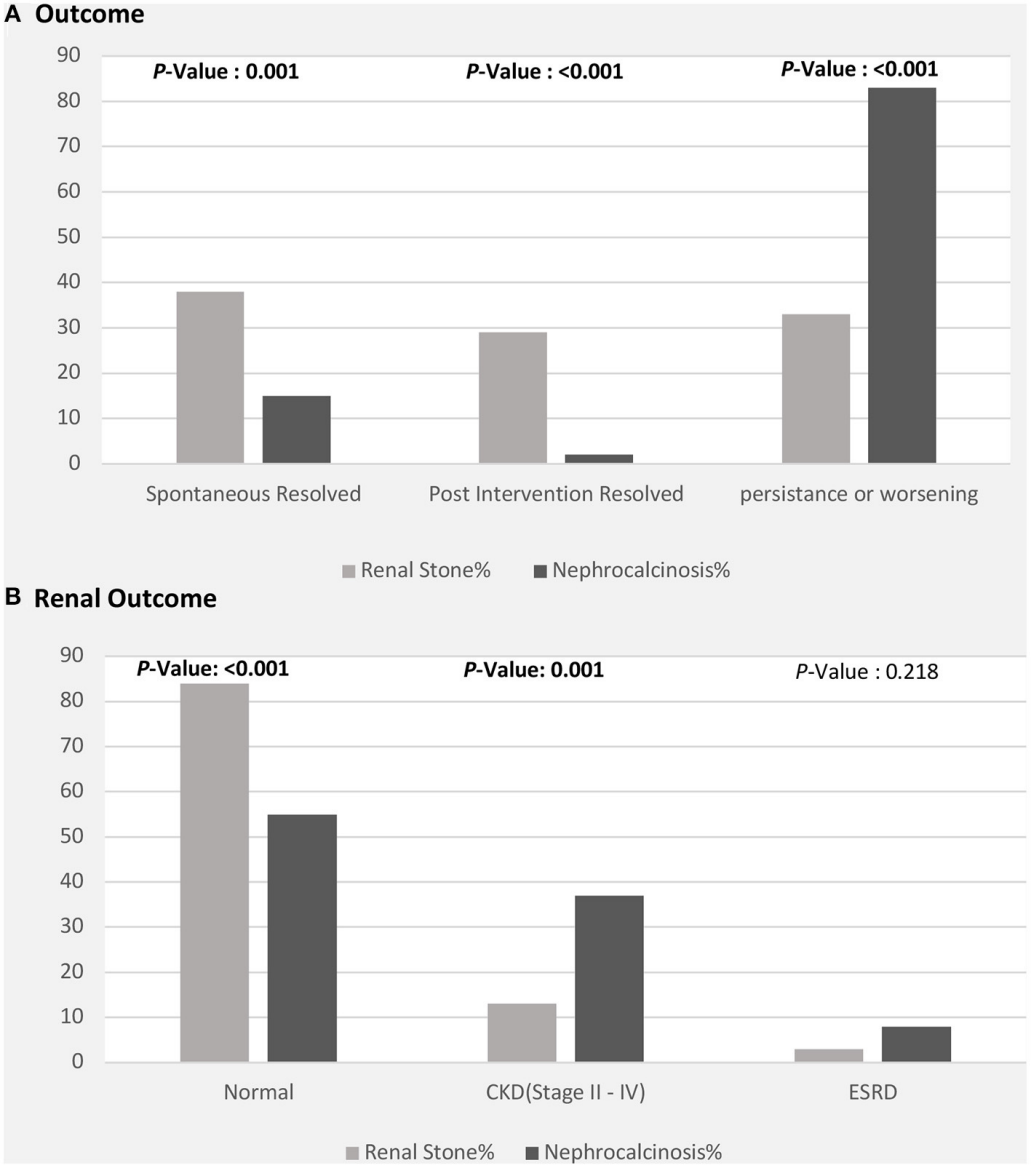
Clinical outcomes among patients with nephrolithiasis/urolithiasis (renal stones) and nephrocalcinosis. CKD, chronic kidney disease; ESRD, end-stage renal disease. **(A)** Radiological outcome in children with nephrolithiasis and nephrocalcinosis. **(B)** Renal function outcome in children with nephrolithiasis and nephrocalcinosis.

The clinical outcomes were significantly better in children with nephrolithiasis; among these, a greater proportion showed spontaneous or post-intervention improvement. In contrast, a greater proportion of patients with nephrocalcinosis showed radiological evidence of worsening or persistent calcinosis (Figure 4A). In addition, patients with nephrocalcinosis progressed more frequently to CKD (stage II-IV) and end-stage kidney disease than those with renal stones (Figure 4B).

## Discussion

Although the regional incidence of kidney stones is high in Saudi Arabia (14), pediatric nephrolithiasis is uncommon. However, since children constitute approximately 40% of the population in Saudi Arabia (15), it is essential to study the epidemiological and clinical features of this disease. Moreover, pediatric nephrolithiasis is associated with significant morbidity, mainly due to the potential for stone recurrence, and consequently should not be overlooked. Unfortunately, few reports have described the epidemiological and clinical features of nephrolithiasis in Saudi pediatric patients. This report describes the epidemiology and underlying causes of pediatric nephrolithiasis and nephrocalcinosis.

According to one study that evaluated urolithiasis in the Middle East, the pattern and etiology of pediatric nephrolithiasis have changed in Saudi Arabia (16). One study reported that pediatric nephrolithiasis constitutes <1% of all kidney stones (13, 17). The mean age at diagnosis was 12 years, with a male-to-female ratio of 2:1. In a single-center study conducted in Jordan, pediatric urolithiasis was reported to constitute 1.85% of all cases of stones (18).

In our report, the mean age at presentation for children with renal stones was 72 months, while the corresponding age for children with nephrocalcinosis was 54 months. Notably, the male-to-female ratio in our patients with nephrolithiasis was 2:1, which is consistent with the findings reported by other investigators (13, 16). In contrast, nephrocalcinosis occurred mainly in female patients, with a male-to-female ratio of 0.66. In a report from Jordan, the mean age of occurrence of pediatric urolithiasis was 14 years, with a male-to-female ratio of 2:1 (18). In another hospital-based study conducted in Iraq, the investigators found that kidney stones occurred at an early age, with most cases diagnosed in children <5 years of age (19). Similar to our study, the authors reported a higher prevalence among men, with a male-to-female ratio of 2.8:1. However; that most papers from the U.S. describe either an equal gender split or higher proportion of female patients in the pediatric stone population (20, 21).

In our study, ~19% of the children with nephrolithiasis presented with signs and symptoms of UTI, which was confirmed with a urine culture in 15% of the cases. A larger proportion of children with nephrocalcinosis presented with symptoms and signs of UTI, which was confirmed in 18% of the cases. In the literature, UTIs were reported in 10–17% of children with urolithiasis (13, 17). The etiology of kidney stones in our study differed from that in a previous report that included 85 children with urolithiasis (13). A-Rasheed et al., in their study, reported that 60% of the children showed idiopathic stones, contrary to the 22% idiopathic cases in our patients with renal stones (13). However, they found that approximately 12% of the children in their study had hypercalciuria, which is similar to the findings in our study. Additionally, a metabolic etiology was implicated in 10% of the children in their study, mainly in the form of cystinuria and primary hyperoxaluria (13). Although metabolic causes were implicated in 69% of our patients, we also found that these were mainly in the form of hyperoxaluria (18%) and cystinuria (18%). These findings suggest the need to perform a metabolic screening test in all children with nephrolithiasis because a UTI, which is a common finding in these children, can mask an underlying metabolic etiology, which may be the primary cause (6).

A strong family history of urolithiasis has been reported in children with kidney stones (19, 22). Although consanguinity has been reported in stone formers, these reports described the findings for adult patients (23, 24). Studies conducted worldwide have shown that pediatric stone formers have a strong family history of urolithiasis. A study conducted at a tertiary center in Brazil reported that 85% of the children in their study had a family history of urolithiasis (25). Furthermore, the investigators found that 83.3% of patients with metabolic changes had a family history of kidney stone disease. In other studies, ~40% of pediatric stone formers had a family history of urolithiasis (26–28). In another study conducted in Iran (29), a family history of urolithiasis was reported in 62.7% of 142 children with kidney stones. We found that 36.3% of the children in our study had a family history of nephrolithiasis and 23.1% had a family history of CKD, confirming the importance of family history in the occurrence of pediatric urolithiasis. A history of consanguinity was found in 64.8% of patients with nephrolithiasis (56% of the patients had parents who were first-degree cousins) and this finding is in agreement with those of previous studies of pediatric nephrolithiasis in Turkey, which indicated that 27-41% of parents had contagious marriages (30).

The clinical outcomes of kidney disease in children are poorly understood. According to a recent report, male and female patients have similar hospitalization rates and frequency of stone episodes (31). In an older study that attempted to investigate clinical outcomes in children with urolithiasis, the investigators were unable to comment on the outcomes of urolithiasis in their patients (28). Although we found that clinical outcomes were better in children with renal stones, children with nephrocalcinosis showed radiological evidence of worsening of the disease. However, we believe that these results only provide preliminary evidence of the disease course in pediatric kidney stone formers so it's might be related to course of the primary disease or risk factors causing either renal stones and nephrocalcinosis.

There are several limitations to this study that merit consideration. One of the main limitations is the retrospective nature of the study and the relatively small sample size. Also we used random urine samples instead of 24 h urine collection as this method more easier for parents and children however known challenges in interpretating random urine data, we used GFR estimation way based on creatinine as it represent the most widely used way, finally there are some deficient data regarding genetic tests and stone analysis for all patients can be explained in view of nature of retrospective study.

## Conclusions

The etiology of nephrolithiasis was identified in the majority of the study population. This was achieved through metabolic screening of all suspected cases of pediatric nephrolithiasis, since metabolic causes were implicated in most of these patients. We also showed that most pediatric stone formers had better clinical outcomes than patients with nephrocalcinosis, which is associated with worse outcomes related to kidney function and disease resolution.

## Data Availability Statement

The raw data supporting the conclusions of this article will be made available by the authors, without undue reservation.

## Ethics Statement

The study design was approved by the Ethical Committees of King Abdulaziz University, Faculty of Medicine, Jeddah, and King Saud University Medical City Hospital, Riyadh, Saudi Arabia, which waived the need to obtain informed consent as it is retrospective data collection. Written informed consent from the participants' legal guardian/next of kin was not required to participate in this study in accordance with the national legislation and the institutional requirements.

## Author Contributions

KA and JK: idea, application for grant, coordination of the study, and writing and editing the manuscript. MS: coordination of the study between three centers and writing and editing the manuscript. AA and M-HT: analysis of the data, writing up the results, and revising the manuscript. ZFZ, ZA, MA, NA, and NK: data collection from both centers and writing and editing the manuscript. All authors have read and approved the manuscript.

## Funding

This project was funded by the Deanship of Scientific Research (DSR) at King Abdulaziz University, Jeddah, under grant no (G:257-140-1439). Also this work was supported by the College of Medicine Research Center, Deanship of Scientific Research, King Saud University, Riyadh, Saudi Arabia. The funding bodies played no role in the design of the study and collection, analysis, and interpretation of data and in writing the manuscript.

## Conflict of Interest

The authors declare that the research was conducted in the absence of any commercial or financial relationships that could be construed as a potential conflict of interest.

## Publisher's Note

All claims expressed in this article are solely those of the authors and do not necessarily represent those of their affiliated organizations, or those of the publisher, the editors and the reviewers. Any product that may be evaluated in this article, or claim that may be made by its manufacturer, is not guaranteed or endorsed by the publisher.
